# Beyond glycemic control: cardiovascular and kidney benefits of GLP-1 receptor agonists and the dual GIP/GLP-1 receptor agonist tirzepatide: a narrative review

**DOI:** 10.3389/fendo.2026.1977433

**Published:** 2026-10-16

**Authors:** Filip Kierzkowski, Wiktoria Skrzypek, Wiesław Bryl, Anna Paczkowska, Karolina Hoffmann

**Affiliations:** 1 Department of Internal Medicine – Ward B, Health Care Complex in Ostrów Wielkopolski, Inpatient Treatment, Ostrów Wielkopolski, Poland; 2 Department and Clinic of Internal Diseases and Metabolic Disorders, Poznan University of Medical Sciences, Poznań, Poland; 3 Department of Pharmacoeconomics and Social Pharmacy, Poznan University of Medical Sciences, Poznań, Poland

**Keywords:** cardiovascular outcomes, cardiovascular-kidney-metabolic syndrome, GLP-1 receptor agonists, kidney outcomes, tirzepatide

## Abstract

Cardiovascular-kidney-metabolic (CKM) syndrome reflects the complex interplay among cardiovascular disease, chronic kidney disease, type 2 diabetes, and obesity. GLP-1 receptor agonists and tirzepatide have emerged as important therapeutic options within CKM syndrome, although the strength and maturity of evidence differ across individual agents and cardiovascular and kidney outcomes. This narrative review summarizes current evidence on the cardiovascular and kidney effects of GLP-1 receptor agonists and the dual GIP/GLP-1 receptor agonist tirzepatide. Large randomized controlled trials have established reductions in major adverse cardiovascular events with several GLP-1 receptor agonists, while dedicated evidence for clinically meaningful chronic kidney disease progression is currently strongest for semaglutide. Cardiovascular benefits of semaglutide have also been demonstrated in individuals with overweight or obesity without type 2 diabetes, supporting effects beyond glucose lowering. Tirzepatide provides substantial improvements in glycemic control and body weight, with expanding evidence supporting cardiovascular benefit, including particularly encouraging findings in obesity-related heart failure with preserved ejection fraction. In contrast, its kidney evidence remains less mature and is derived predominantly from secondary and *post-hoc* analyses rather than from a dedicated kidney outcome trial. Collectively, these findings support a phenotype-oriented and multimodal role for incretin-based therapies in CKM syndrome management while highlighting important differences in the maturity of outcome evidence.

## 1 Introduction

Cardiovascular disease (CVD) and chronic kidney disease (CKD) are among the most clinically important complications associated with type 2 diabetes (T2D) and obesity. These conditions frequently coexist and interact through metabolic, hemodynamic, inflammatory, and vascular pathways, substantially increasing the risk of adverse clinical outcomes and premature mortality (1, 2). Recognition of these interrelationships has led to the concept of CV-kidney-metabolic (CKM) syndrome and to a shift from the management of isolated abnormalities toward integrated cardiovascular (CV), kidney, and metabolic protection (1, 3). This shift is particularly relevant in view of the continuing increase in the prevalence of metabolic disease, driven in part by population aging, low physical activity, poor dietary patterns, and the obesity epidemic (4–7).

Glucagon-like peptide-1 receptor agonists (GLP-1 RAs), initially introduced primarily as glucose-lowering therapies for T2D, have become an important component of this organ-centered therapeutic approach. In addition to improving glycated hemoglobin (HbA1c) and promoting weight reduction (WR), several agents have demonstrated reductions in major adverse CV events (MACE) in patients with T2D and elevated CV risk, together with favorable effects on kidney outcomes (8–13). Early kidney evidence was largely based on secondary or exploratory endpoints, particularly albuminuria, whereas subsequent studies increasingly evaluated clinically meaningful loss of kidney function. At the same time, obesity has emerged not only as a metabolic risk factor but also as a therapeutic context in which incretin-based therapies may provide benefits that are not fully attributable to glucose lowering.

Recent semaglutide trials illustrate this evolution of the evidence. SELECT demonstrated CV benefit in individuals with overweight or obesity and established CVD but without T2D, supporting effects beyond glycemic control (14, 15). FLOW subsequently provided dedicated kidney outcome evidence in patients with T2D and CKD, while SOUL established CV benefit with once-daily oral semaglutide in high-risk T2D (16, 17). Most recently, a prespecified participant-level pooled analysis of SELECT, FLOW, and SOUL, including 30, 787 participants, strengthened the evidence that semaglutide reduces clinically important kidney outcomes across diverse populations with CKM syndrome and across different formulations and diabetes status (18).

The incretin-based therapeutic landscape has also expanded with tirzepatide, a dual glucose-dependent insulinotropic polypeptide (GIP)/GLP-1 RA. The SURPASS and SURMOUNT programs established substantial effects on glycemic control and body weight (BW), while more recent studies have increasingly addressed organ-related outcomes (19, 20). SUMMIT demonstrated benefit in obesity-related heart failure with preserved ejection fraction (HFpEF), and SURPASS-CVOT directly compared tirzepatide with dulaglutide in patients with T2D and established atherosclerotic CVD (21, 22). By contrast, the kidney evidence for tirzepatide remains less mature and is derived mainly from secondary or *post-hoc* analyses, including SURPASS-4, rather than from a dedicated kidney outcome trial (23).

Several recent reviews have summarized important aspects of this rapidly evolving field, including the kidney effects of GLP-1 RAs and tirzepatide, CV and kidney outcomes in CKD, the broader metabolic and organ-related effects of current and next-generation incretin-based therapies, and the CV role of incretin analogues (24–27). The present narrative review is intended to complement rather than duplicate these reports. Its specific added value is a contemporaneous, clinically oriented synthesis of CV and kidney evidence across CKM syndrome that directly juxtaposes the evidence base for single GLP-1 RAs, particularly semaglutide, with that for dual GIP/GLP-1 receptor agonism. In particular, it integrates the transition from exploratory kidney signals to dedicated kidney outcome evidence from FLOW, CV evidence from SELECT and SOUL, tirzepatide outcome data from SUMMIT and SURPASS-CVOT, and the recently reported participant-level pooled kidney analysis of SELECT, FLOW, and SOUL (18). This integrated comparison highlights not only the expanding spectrum of organ protection but also the important difference in the maturity and strength of kidney outcome evidence between semaglutide and tirzepatide.

Important uncertainties nevertheless remain. The magnitude and mechanisms of CV and kidney protection may differ among individual incretin-based therapies, and the extent to which these effects reflect WR, glycemic control, blood pressure (BP) lowering, anti-inflammatory effects, or direct actions on CV and kidney tissues is not fully resolved. Evidence also remains limited in several high-risk or underrepresented populations, including patients with advanced CKD or receiving dialysis, older and frail individuals, and people with type 1 diabetes (T1D).

Accordingly, this narrative review aims to critically synthesize current evidence on the CV and kidney effects of GLP-1 RAs and tirzepatide within an integrated CKM syndrome framework. Particular emphasis is placed on the evolution from CV safety and exploratory kidney endpoints toward dedicated and clinically meaningful cardiorenal outcomes, on differences in the strength of evidence between single and dual incretin-based therapies, and on the translational and evidence gaps that should guide future clinical research.

## 2 Methods and scope of the review

This narrative review was prepared on the basis of a focused literature search followed by expert interpretation of the available evidence. PubMed/MEDLINE, Web of Science, Embase, Google Scholar, and Scopus were initially searched from database inception through 15 July 2026. During revision, a targeted literature update through 31 August 2026 was performed to capture newly published pivotal outcome studies, clinically oriented reviews, guideline documents, and safety analyses relevant to the scope of the review. Search terms included combinations of “glucagon-like peptide-1 receptor agonist”, “dual GIP/GLP-1 receptor agonist”, “tirzepatide”, “semaglutide”, “dulaglutide”, “liraglutide”, “CV outcomes”, “kidney outcomes”, “cardio-kidney-metabolic disease”, “cardio-kidney-metabolic syndrome”, “obesity”, “type 2 diabetes”, “incretin”, “chronic kidney disease”, “cardiovascular disease”, “CV events”, “renal effects”, and “renal outcomes”.

Priority was given to randomized controlled trials (RCTs), prospective cohort studies, international recommendations and guidelines, clinical reviews, systematic reviews, and meta-analyses. Relevant publications were also identified through manual screening of the reference lists of key articles. The literature update further encompassed studies addressing potential mechanisms of CV and kidney protection, combination treatment strategies, safety and tolerability, emerging incretin-based therapies, and major challenges associated with their implementation.

As the primary aim of this review was to provide a clinically oriented synthesis of current knowledge and highlight important translational gaps rather than quantitatively combine study results, a formal risk-of-bias assessment and meta-analysis were not undertaken. Accordingly, this work should be regarded as a narrative review rather than a systematic review or meta-analysis.

## 3 GLP-1 and GIP receptors: biological basis for cardiorenal effects

GLP-1 is an incretin hormone secreted predominantly by enteroendocrine L cells, which are abundant in the distal small intestine and colon. Its secretion increases rapidly after nutrient intake, particularly in response to carbohydrates and fats. GLP-1 enhances glucose-dependent insulin secretion, suppresses glucagon release under hyperglycemic conditions, delays gastric emptying, and promotes satiety, thereby contributing to postprandial glucose homeostasis and energy balance (28).

The second major incretin hormone, glucose-dependent insulinotropic polypeptide (GIP), is secreted predominantly by enteroendocrine K cells located in the duodenum and proximal jejunum. Its release is stimulated by nutrient ingestion, particularly glucose and dietary fat, and GIP subsequently enhances insulin secretion from pancreatic β-cells in a glucose-dependent manner (29–31).

Native GLP-1 and GIP have short biological half-lives because they are rapidly degraded by dipeptidyl peptidase-4 (DPP-4) (32). This limited stability has driven the development of pharmacological incretin-based therapies with substantially prolonged activity, including degradation-resistant GLP-1 RAs and, more recently, dual GIP/GLP-1 RA.

The biological effects of GLP-1 extend well beyond pancreatic β-cell stimulation. GLP-1 receptor (GLP-1R) signaling has been identified in several tissues involved in metabolic and CV regulation, including the pancreas, gastrointestinal (GI) tract, central nervous system (CNS), and CV system. However, the precise cellular distribution of GLP-1R in some organs, particularly the human kidney, remains incompletely defined and should be interpreted cautiously (33). This broad pattern of receptor-mediated and indirect systemic actions provides a biological basis for the pleiotropic effects of GLP-1 RAs beyond glucose lowering.

Activation of central GLP-1R signaling enhances satiety and reduces energy intake, while delayed gastric emptying may further contribute to reduced caloric intake (34). The resulting WR is accompanied by improvements in several cardiometabolic risk factors, including BP, glycemic control, and lipid metabolism (35). In addition, experimental and clinical evidence suggests that GLP-1 RAs exert anti-inflammatory, antioxidative, and vascular effects that may improve endothelial function and attenuate fibrosis and cellular injury (36, 37). These direct and indirect mechanisms are increasingly considered relevant contributors to the CV benefits observed with GLP-1 RA therapy (37).

Several complementary mechanisms may contribute to kidney protection. GLP-1R activation promotes natriuresis, partly through modulation of sodium transport in the proximal tubule, and may influence kidney hemodynamics and glomerular hyperfiltration. Furthermore, experimental studies suggest reductions in oxidative stress, inflammation, endothelial dysfunction, and fibrotic signaling (38, 39). Nevertheless, the exact contribution of direct kidney GLP-1R signaling remains uncertain, as GLP-1R expression in the human kidney appears to be more restricted than previously assumed. Therefore, the kidney effects of GLP-1 RAs are likely to result from a combination of direct and indirect mechanisms, including improvements in glycemic control, BW, BP, vascular function, inflammation, and kidney hemodynamics (39).

When considering incretin receptor pharmacology, tirzepatide occupies a distinct position as a single peptide with dual agonistic activity at both GIP and GLP-1 receptors (40). Pharmacological studies have demonstrated an imbalanced signaling profile, with greater engagement of GIPR than GLP-1R and distinct signaling properties at the GLP-1R (41). GIPRs are expressed in pancreatic β-cells as well as in several extra-pancreatic tissues, including adipose tissue and regions of the CNS involved in metabolic and appetite regulation (42). Simultaneous activation of GIPR and GLP-1R may therefore produce complementary effects on glucose regulation, energy intake, and BW (40). This dual-receptor mechanism provides a biological rationale for the pronounced improvements in glycemic control and BW observed with tirzepatide and has stimulated increasing interest in whether these metabolic effects may also translate into CV and kidney protection (24, 43).

**Table 1** summarizes the key characteristics, tissue distribution, and physiological roles of GIPRs and GLP-1Rs in metabolic and cardiorenal regulation (44–59).

**Table 1:** Biology of GIP and GLP-1 receptors.

Feature	Hormone-secreting cells	Stimulating hormone secretion	Receptor localization	Effect on pancreatic β-cells and glucagon secretion	Effect on gastric emptying	Effect on adipose tissue	Impact on the heart	Effect on the kidneys
GLP-1 Receptor	L-cells located primarily in the ileum and colon	Glucose, fats, proteins, and bile acids released after a meal	Pancreatic β-cells, CNS neurons (particularly the hypothalamus and brainstem), stomach, heart, blood vessels, kidneys (mainly arterioles and selected segments of the nephron), lungs	It increases glucose-dependent insulin secretion, inhibits glucagon secretion under hyperglycemic conditions	It significantly delays gastric emptying	GLP-1 receptor agonism indirectly reduces body fat primarily by suppressing appetite and energy intake	It reduces ischemia-reperfusion injury and improves endothelial function and left ventricular function	Stimulation of the GLP-1 receptor increases natriuresis.
GIP Receptor	K cells, located primarily in the duodenum and the proximal part of the jejunum	Mainly glucose and long-chain FA after a meal, as well as amino acids.	Pancreatic β-cells, adipocytes, the CNS, bone (osteoblasts and osteoclasts), the adrenal glands, and cardiac muscle	It enhances glucose-dependent insulin secretion. It stimulates glucagon secretion during hypoglycemia.	It does not have a significant effect on the rate of gastric emptying	GIPR activation enhances insulin signaling and promotes FA uptake and lipid storage in adipose tissue	GIPR are expressed in cardiomyocytes and regulate cardiac lipid metabolism, including myocardial TG storage and fatty-acid oxidation	The direct effects of GIPR stimulation in the kidneys have not been thoroughly investigated; potential renal benefits are likely primarily indirect and associated with improved systemic metabolic control
Reference	(44)	(45, 46)	(33, 47)	(48, 49)	(50–52)	(47, 53)	(54–57)	(58, 59)

CNS, central; FA, fatty acids; GIP - glucose-dependent insulinotropic polypeptide; GLP-1 - glucagon-like peptide-1; R-receptor; RA - receptor agonist; TG, triglyceride.

## 4 Evidence from randomized controlled trials

### 4.1 Single GLP-1 RAs

Early evidence suggesting kidney benefits of GLP-1 RAs emerged predominantly from CV outcome trials (CVOTs) that were designed primarily to assess CV safety and efficacy rather than CKD progression. Consequently, kidney findings from these trials should be interpreted according to the status and composition of the respective endpoints. In particular, most early kidney outcomes were prespecified secondary or exploratory composites that included albuminuria, a relatively frequent event, together with less frequent outcomes reflecting loss of kidney function or kidney failure.

In LEADER, liraglutide reduced the risk of the prespecified secondary composite kidney outcome, which included new-onset persistent macroalbuminuria, persistent doubling of serum creatinine, kidney failure requiring kidney replacement therapy (KRT), or death due to kidney disease (12). However, the overall benefit was driven primarily by a reduction in new-onset persistent macroalbuminuria (HR 0.74, 95% CI 0.60-0.91), whereas the risks of persistent doubling of serum creatinine and end-stage kidney disease did not differ significantly between treatment groups. Thus, LEADER provided an important early signal of kidney benefit but did not establish an effect on kidney failure as a primary outcome.

Similarly, the kidney analysis of REWIND was exploratory. Dulaglutide reduced the composite kidney outcome comprising new macroalbuminuria, a sustained ≥30% decline in eGFR, or chronic KRT (HR 0.85, 95% CI 0.77-0.93). The clearest effect was observed for new macroalbuminuria (HR 0.77, 95% CI 0.68-0.87), whereas the ≥30% sustained decline in eGFR (HR 0.89, 95% CI 0.78-1.01) and chronic KRT (HR 0.75, 95% CI 0.39-1.44) did not individually reach statistical significance (13). These findings therefore support a favorable effect on albuminuria and suggest possible attenuation of CKD progression but provide less definitive evidence regarding hard kidney outcomes.

HARMONY Outcomes demonstrated CV benefit with albiglutide in patients with T2D and established CVD, but it was not a dedicated kidney outcome trial and its primary publication did not establish a specific kidney-protective effect (11). Kidney-protective conclusions regarding albiglutide should therefore remain cautious.

AMPLITUDE-O also primarily addressed CV outcomes, with the kidney composite included as a secondary outcome. Efpeglenatide reduced a composite kidney outcome that incorporated incident macroalbuminuria together with sustained deterioration in eGFR and kidney failure-related components (HR 0.68, 95% CI 0.57-0.79) (60). However, the kidney-function-focused composite excluding albuminuria was less conclusive. Accordingly, AMPLITUDE-O strengthens the overall signal of kidney benefit associated with GLP-1 RA therapy but does not provide the same level of evidence as a trial specifically powered for clinically meaningful kidney disease progression.

**Table 2** summarizes the key characteristics and principal findings of the original RCTs described above. 

**Table 2:** Key characteristics and findings of the LEADER, REWIND, HARMONY outcomes, and AMPLITUDE-O trials.

Clinical trial acronym	LEADER	REWIND	HARMONY Outcomes	Amplitude-O
Article title, year, (ref)	Liraglutide and Cardiovascular Outcomes in Type 2 Diabetes, 2016 (8)	Dulaglutide and cardiovascular outcomes in type 2 diabetes (REWIND), 2019 (10)	Albiglutide and cardiovascular outcomes in patients with type 2 diabetes and cardiovascular disease (Harmony Outcomes), 2018 (11)	Cardiovascular and Renal Outcomes with Efpeglenatide in Type 2 Diabetes, 2021 (60)
Study type	RCT, double-blind	RCT, double-blind	RCT, double-blind	RCT, double-blind
Study population	Patients with T2D and high CV risk	Patients with T2D and either established CVD or multiple CV risk factors	Patients with T2D and established CVD	Patients with T2D and established CVD or CKD plus ≥1 additional CV risk factor
Number of randomized participants (n)	9340	9901	9463	4076
Mean age of participants (y.)	64.3	66.2	64.1	64.5
Baseline HbA1c [%]	8.7	7.2	8.7	8.9
Baseline eGFR [mL/min/1.73 m²]	80.4 ± 27.3	Not reported in the main publication	79.0	72.4
Baseline BMI [kg/m²]	32.5 ± 6.3	32.3	32.3	32.7
Intervention	Liraglutide 1.8 mg once per day (s.c.)	Dulaglutide 1.5 mg once per week, s.c.	Albiglutide 30–50 mg once per week, s.c.	Efpeglenatide 4 mg or 6 mg once per week, s.c.
Follow-up duration -median [y.]	3.8	5.4	1.6	1.81
Primary endpoint	CV death, non-fatal MI, non-fatal stroke	CV death, non-fatal MI, non-fatal stroke	CV death, non-fatal MI, non-fatal stroke	CV death, non-fatal MI, non-fatal stroke
Primary endpoint result	HR 0.87 (95% CI 0.78–0.97)	HR 0.88 (95% CI 0, 79–0, 99)	HR 0.78 (95% CI 0.68–0.90)	HR 0.73 (95% CI 0.58–0.92)
Death from CV causes	HR 0.78 (95% CI 0.74-0.97)	Not reported as a separate numerical result in the main publication	Not reported as a separate numerical result in the main publication	Not reported as a separate numerical result in the main publication
Non-fatal MI	HR 0.86 (95% CI 0.73–1.00)	Not reported as a separate numerical result in the main publication	Not reported as a separate numerical result in the main publication	Not reported as a separate numerical result in the main publication
Non-fatal stroke	HR 0.88 (95% CI 0.75–1.03)	Not reported as a separate numerical result in the main publication	Not reported as a separate numerical result in the main publication	Not reported as a separate numerical result in the main publication
Kidney endpoint: status and definition	Prespecified secondary composite: new persistent macroalbuminuria, persistent doubling of serum creatinine, kidney failure requiring KRT, or kidney-related death (12)	Exploratory composite: new macroalbuminuria, sustained ≥30% decline in eGFR, or chronic KRT (13)	No dedicated kidney outcome in the main CVOT publication	Secondary composite including incident macroalbuminuria, sustained ≥40% eGFR decline, KRT, or sustained eGFR <15 mL/min/1.73 m² (60)
Kidney outcome: result and interpretation	HR 0.78 (95% CI 0.67-0.92); benefit driven mainly by macroalbuminuria (HR 0.74); doubling of serum creatinine and kidney failure were not significantly reduced	HR 0.85 (95% CI 0.77-0.93); strongest effect for macroalbuminuria (HR 0.77); sustained eGFR decline and chronic KRT were not individually significant	No dedicated kidney-protective effect established	HR 0.68 (95% CI 0.57-0.79); kidney-function-focused composite excluding albuminuria was less conclusive

BMI, body mass index; CKD, chronic kidney disease; CV, cardiovascular; CVD, cardiovascular disease; CVOT, cardiovascular outcome trial; eGFR, estimated glomerular filtration rate; HbA1c, glycated hemoglobin; MI, myocardial infarction; s.c., subcutaneously; T2D, type 2 diabetes; y., years.

#### 4.1.1 Semaglutide

SUSTAIN-6 was primarily designed as a CV safety trial in patients with T2D at high CV risk rather than as a dedicated kidney outcome study. The primary composite CV outcome of CV death, non-fatal myocardial infarction (MI), or non-fatal stroke occurred less frequently with semaglutide than with placebo (HR 0.74, 95% CI 0.58-0.95) (9). Among the individual components, the incidence of non-fatal stroke was lower with semaglutide (HR 0.61, 95% CI 0.38-0.99), whereas differences in CV death and non-fatal MI were not statistically significant. The prespecified secondary microvascular outcome of new or worsening nephropathy was also reduced (HR 0.64, 95% CI 0.46-0.88), but this benefit was driven predominantly by persistent macroalbuminuria, while events reflecting more advanced kidney disease were infrequent and were not significantly reduced. Accordingly, the SUSTAIN-6 kidney findings should be interpreted as supportive rather than definitive evidence of prevention of clinically advanced CKD progression (9). A subsequent *post-hoc* analysis reported that CV and kidney treatment effects were broadly consistent across baseline BMI categories (61).

SELECT subsequently demonstrated CV benefit with once-weekly semaglutide 2.4 mg in individuals with overweight or obesity and established CVD but without diabetes. Semaglutide reduced the primary MACE outcome compared with placebo (HR 0.80, 95% CI 0.72-0.90) (14). A prespecified kidney analysis also identified a lower risk of a composite kidney outcome (HR 0.78, 95% CI 0.63-0.96), but SELECT was a CV outcomes trial rather than a dedicated kidney trial (15). The absence of diabetes demonstrates that glucose lowering is not required for CV benefit in this population, although it does not establish independence from weight loss or other metabolic and hemodynamic changes.

Additional evidence regarding the clinical effects of semaglutide in obesity-related HFpEF comes from the STEP-HFpEF and STEP-HFpEF DM trials. In the prespecified pooled analysis of 1, 145 participants, semaglutide improved HF-related symptoms and physical limitations and reduced BW compared with placebo. At week 52, the mean between-group difference in the Kansas City Cardiomyopathy Questionnaire Clinical Summary Score was 7.5 points (95% CI 5.3-9.8), while the mean between-group difference in BW was -8.4% (95% CI -9.2 to -7.5). Semaglutide also improved exercise capacity, with a 17.1-m greater increase in 6-minute walk distance (95% CI 9.2-25.0), and reduced C-reactive protein concentrations (treatment ratio 0.64, 95% CI 0.56-0.72) compared with placebo (62). These findings demonstrate clinically relevant improvements in obesity-related HFpEF, including in patients without T2D, but the trials were not designed to establish the specific biological mechanisms responsible for these clinical benefits.

FLOW provided pivotal dedicated kidney outcome evidence for semaglutide in patients with T2D and CKD (16). The primary composite outcome comprised kidney failure, a sustained ≥50% reduction in eGFR from baseline, or death from kidney-related or CV causes. Semaglutide reduced the risk of this composite outcome by 24% compared with placebo (HR 0.76, 95% CI 0.66-0.88). Importantly, this finding should not be interpreted as evidence of an independent reduction in kidney-related mortality, as death from kidney-related causes was not significantly reduced when considered as a standalone endpoint. CV mortality was lower with semaglutide (HR 0.71, 95% CI 0.56-0.89), and all-cause mortality was also reduced (HR 0.80, 95% CI 0.67-0.95). In addition, the mean annual decline in eGFR was 1.16 mL/min/1.73 m²/year less steep in the semaglutide group than in the placebo group (P<0.001), providing further evidence of slower loss of kidney function.

The kidney evidence for semaglutide has been further strengthened by a prespecified participant-level pooled analysis of 30, 787 participants from SELECT, FLOW, and SOUL (18). The populations differed substantially in baseline risk and semaglutide regimen: FLOW enrolled patients with T2D and CKD receiving once-weekly subcutaneous semaglutide 1.0 mg, SELECT enrolled individuals with overweight or obesity and established CVD without diabetes receiving 2.4 mg weekly, and SOUL enrolled patients with T2D and established atherosclerotic CVD, CKD, or both receiving oral semaglutide 14 mg daily. The harmonized primary kidney composite comprised persistent ≥50% reduction in eGFR, kidney failure defined as persistent eGFR <15 mL/min/1.73 m² or initiation of KRT, kidney-related death, or CV-related death. Semaglutide reduced this outcome by 16% (HR 0.84, 95% CI 0.77-0.91). A narrower secondary composite excluding CV-related death was also reduced (HR 0.80, 95% CI 0.69-0.92), indicating that the overall association was not dependent on CV death. The pooled analysis broadens the evidence across CKM syndrome phenotypes but complements rather than replaces FLOW as the dedicated kidney outcome trial and does not establish the biological mechanisms underlying the observed benefit.

SEMPA provided mechanistic rather than hard outcome evidence regarding combined semaglutide and empagliflozin therapy. Combination treatment did not produce a significant additional reduction in vascular stiffness, although improvements in 24-hour BP and time in range were observed (63). These intermediate findings should be distinguished from evidence regarding major CV or kidney events. SOUL subsequently established CV benefit with once-daily oral semaglutide in patients with T2D and established atherosclerotic CVD, CKD, or both (HR 0.86, 95% CI 0.77-0.96 for MACE) (17).

**Table 3** summarizes the key characteristics and findings of clinical studies evaluating semaglutide. 

**Table 3:** Key characteristics and findings of clinical studies evaluating semaglutide.

Clinical trial acronym	SUSTAIN-6	SELECT	FLOW	SEMPA	SOUL
Article title, year, (ref)	Semaglutide and Cardiovascular Outcomes in Patients with Type 2 Diabetes, 2016 (9),	Semaglutide and Cardiovascular Outcomes in Obesity without Diabetes, 2023 (14)	Effects of Semaglutide on Chronic Kidney Disease in Patients with Type 2 Diabetes, 2024 (16),	Separate and combined effects of empagliflozin and semaglutide on vascular function, 2024 (63),	Oral Semaglutide and Cardiovascular Outcomes in High-Risk Type 2 Diabetes, 2025 (17),
Study type	Phase 3 RCT; cardiovascular safety trial	Randomized, double-blind, placebo-controlled cardiovascular outcomes trial	Phase 3 RCT; dedicated kidney outcomes trial	Randomized mechanistic trial	Phase 3b randomized, double-blind, placebo-controlled cardiovascular outcomes trial
Study population	Patients with T2D and high CV risk	Patients with overweight or obesity (BMI ≥27 kg/m²) and established CVD, without T2D	Patients with T2D and CKD	Patients with T2D and increased cardio-renal risk	Patients with T2D and established atherosclerotic CVD, CKD or both
Number of randomized participants (n)	3297	17604	3533	120	9650
Mean age of participants [y.]	64.6 ± 7.4	61.6 ± 8.9	66.6 ± 9.0	Not reported as a single value	66.0 ± 7.5
Baseline HbA1c [%]	8.7 ± 1.5	5.8 ± 0.4	7.8 ± 1.3	Reported separately by treatment group	8.0 ± 1.5
Baseline eGFR [mL/min/1.73 m²]	74.0 ± 21.0	Not a prespecified kidney population characteristic	47.0 ± 15.0	Reported separately by treatment group	Reported in baseline characteristics
Baseline BMI [kg/m²]	32.8 ± 6.2	33.3 ± 5.0	32.0 ± 6.4	Reported separately by treatment group	31.1 ± 5.7
Intervention	Semaglutide 0.5 mg or 1.0 mg once per week (s.c.)	Semaglutide 2.4 mg once per week (s.c.)	Semaglutide 1.0 mg once per week (s.c.)	Empagliflozin 10 mg once per day or Semaglutide 1 mg once per week or both Semaglutide 1 mg once per week + empagliflozin 10 mg once per day	Semaglutide at a dose of up to 14 mg once per day (p.o.)
Follow-up duration	Median 2.1 years	Mean 39.8 months	Median 3.4 years	32 weeks	Median 47.5 months
Primary endpoint	CV death, non-fatal MI, non-fatal stroke	CV death, non-fatal MI, non-fatal stroke	Kidney failure, sustained ≥50% reduction in eGFR, or death from kidney-related or cardiovascular causes	vascular function (cfPWV) and renal parameters	CV death, non-fatal MI, non-fatal stroke
Primary endpoint result	HR 0.74 (95% CI 0.58–0.95)	HR 0.80 (95% CI 0.72–0.90)	HR 0.76 (95% CI 0.66–0.88)	–	HR 0.86 (95% CI 0.77–0.96)
Death from cardiovascular causes	HR 0.98 (95% CI 0.65-1.48)	HR 0.85 (95% CI 0.71 do 1.01)	HR 0.71 (95% CI 0.56-0.89)	–	HR 0, 80 (95% CI 0, 62–1, 02)
Non-fatal myocardial infarction	HR 0.74 (95% CI 0.51–1.08)	HR 0.72 (95% CI 0.61–0.85)	–	–	–
Non-fatal stroke	HR 0.61 (95% CI 0.38–0.99)	HR 0.93 (95% CI 0.74–1.15)	–	–	–
Kidney endpoint: status and definition	Prespecified secondary microvascular outcome: new or worsening nephropathy, including persistent macroalbuminuria and kidney-function components	Prespecified kidney analysis of a cardiovascular outcomes trial; not a dedicated kidney trial (15)	Primary dedicated kidney composite: kidney failure, sustained ≥50% eGFR reduction, or kidney-related or cardiovascular death (16)	Mechanistic/intermediate kidney parameters; no hard kidney outcome trial	Cardiovascular outcomes trial; kidney evidence derived from secondary and pooled analyses rather than a primary kidney endpoint
Kidney outcome: result and interpretation	HR 0.64 (95% CI 0.46-0.88); predominantly driven by persistent macroalbuminuria; no definitive reduction in kidney failure established	HR 0.78 (95% CI 0.63-0.96); supportive kidney signal in a CVOT population without diabetes	Primary composite HR 0.76 (95% CI 0.66-0.88); kidney-specific components HR 0.79 (95% CI 0.66-0.94); annual eGFR decline 1.16 mL/min/1.73 m²/year less steep; CV death HR 0.71 and all-cause death HR 0.80	Intermediate outcomes only; should not be interpreted as evidence of hard kidney protection	No standalone dedicated kidney outcome result; contributes to broader pooled evidence

BMI, body mass index; BW, body weight; cfPWV, carotid-femoral pulse wave velocity; CKD, chronic kidney disease; CV, cardiovascular; CVD, cardiovascular disease; eGFR, estimated glomerular filtration rate; ESKD, end-stage kidney disease; HbA1c, glycated hemoglobin; MI, myocardial infarction; p.o., per os; s.c., subcutaneously; T2D, type 2 diabetes; y., years; WR, weight reduction. A prespecified participant-level pooled analysis of SELECT, FLOW, and SOUL (18) reported HR 0.84 (95% CI 0.77-0.91) for the harmonized kidney composite and HR 0.80 (95% CI 0.69-0.92) when cardiovascular death was excluded.

### 4.2 Dual GIP/GLP-1 RAs

#### 4.2.1 Tirzepatide

Building on the evidence established for single GLP-1 RAs, tirzepatide has progressively evolved from a highly effective glucose- and weight-lowering therapy toward a broader cardiometabolic intervention. Recent clinically oriented syntheses have proposed considering its evidence across a CV continuum encompassing metabolic risk-factor modification, obesity, kidney outcomes, atherosclerotic CVD, and obesity-related HFpEF (64). Within this framework, the SURPASS program established substantial improvements in glycemic control, BW, and multiple cardiometabolic risk factors in patients with T2D, while the SURMOUNT program extended these findings to obesity and obesity-related complications. An overview of the major SURMOUNT trials is presented in **Table 4**. More recent outcome trials have expanded the evidence base further: SURPASS-CVOT directly evaluated MACE against dulaglutide in patients with T2D and established atherosclerotic CVD, whereas SUMMIT evaluated obesity-related HFpEF. This progression provides a useful clinical framework for interpreting the individual tirzepatide trials.

**Table 4:** Key characteristics and findings of the SURMOUNT-1–5 and SURMOUNT-OSA trials.

Clinical trial acronym	Study population	Primary objective	Intervention	Control group	Key findings	Reference
SURMOUNT-1	Adults with obesity or overweight, without T2D	To evaluate efficacy and safety of tirzepatide for chronic weight management	Tirzepatide 5 mg, 10 mg, or 15 mg once per week	Placebo	Tirzepatide 15 mg resulted in a 20.9% mean reduction in BW compared with 3.1% with placebo; approximately 57% achieved ≥20% WR after 72 weeks.	(20)
SURMOUNT-2	Adults with obesity or overweight and T2D	To evaluate efficacy and safety of tirzepatide for WR in patients with T2D	Tirzepatide 10 mg or 15 mg once per week	Placebo	Tirzepatide produced a 14.7% mean reduction in BW (15 mg) compared with 3.2% with placebo and significantly improved HbA1c.	(65)
SURMOUNT-3	Adults with obesity or overweight who achieved ≥5% WR during a 12-week intensive lifestyle intervention	To determine whether tirzepatide provides additional WR after lifestyle-induced WR	Tirzepatide 10 mg or 15 mg once per week	Placebo	Participants receiving tirzepatide achieved an additional 18.4% reduction in BW, whereas placebo was associated with weight regain.	(66)
SURMOUNT-4	Adults with obesity or overweight after 36 weeks of tirzepatide treatment	To evaluate maintenance of weight loss with continued tirzepatide versus treatment withdrawal	Tirzepatide 10 mg or 15 mg once per week	Placebo after 36 weeks of tirzepatide	Continued tirzepatide resulted in a total mean WR of 25.3%, whereas switching to placebo led to partial weight regain.	(74)
SURMOUNT-5	Adults with obesity or overweight without T2D	To compare tirzepatide with semaglutide for chronic weight management	Tirzepatide 10 mg or 15 mg once per week	Semaglutide 1.7 mg or 2.4 mg once per week	Tirzepatide achieved greater WR than semaglutide (20.2% vs 13.7%), with more participants achieving ≥20% and ≥25% WR.	(67)
SURMOUNT-OSA	Adults with obesity and moderate-to-severe OSA, with or without PAP therapy	To evaluate effects of tirzepatide on OSA severity and BW	Tirzepatide up to 15 mg once per week	Placebo	Tirzepatide significantly reduced AHI, BW, and hypoxic burden and improved sleep-related outcomes.	(68)

AHI, apnea-hypopnea index; BW, body weight; OSA, obstructive sleep apnea; PAP, positive airway pressure; T2D, type 2 diabetes; y., years; WR, weight reduction.

Across the SURPASS program, tirzepatide produced substantial reductions in HbA1c and BW. SURPASS-2 demonstrated greater reductions in both outcomes with tirzepatide than with semaglutide 1.0 mg (19), while SURPASS-3 and SURPASS-5 demonstrated efficacy when tirzepatide was used with background glucose-lowering therapy, including SGLT2 inhibitors or basal insulin (69, 70). SURPASS-1 established efficacy versus placebo in patients treated with diet and exercise alone, while SURPASS-4 confirmed substantial glycemic and weight effects versus insulin glargine in patients with increased CV risk (71, 72). SURPASS-CVOT subsequently compared tirzepatide directly with dulaglutide in patients with T2D and established atherosclerotic CVD. Tirzepatide was non-inferior, but not statistically superior, to dulaglutide for MACE (HR 0.92, 95.3% CI 0.83-1.01), despite greater reductions in BW and HbA1c (22, 73). These findings support an expanding CV evidence base while also illustrating that greater metabolic efficacy does not necessarily translate proportionally into greater reduction in hard CV outcomes.

The SURMOUNT program demonstrated marked and sustained WR with tirzepatide in adults with obesity or overweight, including people with and without T2D and those undergoing lifestyle intervention or treatment withdrawal (20, 65–68, 74). In SUMMIT, tirzepatide reduced the composite risk of CV death or worsening HF in patients with obesity-related HFpEF and improved health status and physical limitations (21). The treatment effect on the composite was driven primarily by fewer worsening HF events rather than by a demonstrated standalone reduction in CV mortality. These results are particularly relevant to the obesity-related HFpEF phenotype and should be distinguished from evidence regarding atherosclerotic MACE or CKD progression.

Regarding kidney effects, the evidence for tirzepatide is derived primarily from secondary and *post-hoc* analyses rather than from a dedicated kidney outcome trial. In the *post-hoc* kidney analysis of SURPASS-4, tirzepatide was associated with a slower decline in eGFR, reduced albuminuria, and a lower risk of the composite kidney endpoint compared with insulin glargine (23). A separate *post-hoc* analysis using cystatin C-based estimates supported a slower decline in kidney function (75). These findings provide supportive evidence of a potential kidney benefit but should be interpreted cautiously because SURPASS-4 was not designed or powered primarily to evaluate CKD progression. In contrast, semaglutide has dedicated kidney outcome evidence from FLOW and additional pooled evidence across SELECT, FLOW, and SOUL (16, 18, 76).

Tirzepatide has also demonstrated benefits in other obesity-related organ disease, including histological improvement in metabolic dysfunction-associated steatohepatitis in SYNERGY-NASH and related exploratory analyses (77, 78). These outcomes are clinically relevant but remain outside the primary CV and kidney focus of the present review.

## 5 Discussion

The available evidence supports a fundamental shift in the therapeutic positioning of incretin-based therapies. GLP-1 RAs are no longer regarded solely as glucose-lowering or weight-management agents within a predominantly glucocentric treatment paradigm. Instead, CV and kidney outcome data have contributed to a more comprehensive, organ-centered approach to CKM syndrome management. At the same time, the strength of evidence differs materially among individual agents and outcomes: CV benefit has been established for several GLP-1 RAs, dedicated kidney outcome evidence is currently strongest for semaglutide, and the kidney evidence for tirzepatide remains predominantly secondary and *post-hoc* (8–18, 21–23).

### 5.1 Interpreting kidney outcomes across trials

An important consideration when interpreting the kidney evidence is that not all kidney outcomes provide the same level of clinical evidence. Albuminuria is a well-established marker of kidney and CV risk and its reduction may indicate favorable kidney effects; however, improvement in albuminuria should not be considered equivalent to prevention of progressive loss of kidney function or kidney failure. This distinction is particularly important for the early GLP-1 RA CVOTs. In LEADER, REWIND, and SUSTAIN-6, favorable composite kidney outcomes were largely influenced by reductions in new-onset or persistent macroalbuminuria, whereas events reflecting advanced kidney disease, such as substantial sustained eGFR loss or kidney replacement therapy (KRT), were substantially less frequent and the individual trials were generally not powered to demonstrate treatment effects on these components (9, 12, 13). Similarly, although AMPLITUDE-O demonstrated a significant reduction in a secondary composite kidney outcome, the composite incorporated albuminuria together with kidney-function endpoints, and evidence for the latter considered separately was less conclusive (60).

Composite kidney endpoints also require careful interpretation because they combine outcomes of different clinical severity and frequency. Harder kidney outcomes - including sustained clinically meaningful reductions in eGFR, progression to kidney failure, and the need for KRT - provide more direct evidence of modification of CKD progression. Mortality outcomes should also be considered separately: reductions in CV or all-cause mortality are clinically highly relevant but should not, by themselves, be interpreted as direct evidence of kidney protection. FLOW is qualitatively different from the earlier CVOT evidence because CKD progression was the primary focus of the trial. Although death from kidney-related or CV causes was included in the primary composite endpoint, semaglutide also reduced the composite restricted to kidney-specific components (HR 0.79, 95% CI 0.66-0.94) and slowed annual eGFR decline (16). The SELECT-FLOW-SOUL pooled analysis further supports consistency of semaglutide-associated kidney benefit across heterogeneous CKM syndrome populations, including after CV death was excluded from the composite, but it complements rather than replaces FLOW and does not remove heterogeneity related to diabetes status, baseline kidney function, formulation, dose, and CV risk (18). FLOW therefore remains the principal dedicated kidney outcome evidence, whereas favorable kidney findings with tirzepatide currently remain supportive and hypothesis-generating (23).

### 5.2 Weight loss versus other mediators of cardiorenal protection

A central unresolved question is the extent to which CV and kidney benefits of incretin-based therapies are mediated by WR versus other metabolic, hemodynamic, inflammatory, or potentially direct tissue effects. Weight loss itself may plausibly improve cardiorenal risk through reductions in BP and visceral adiposity, improvements in insulin resistance and glycemic control, attenuation of systemic inflammation, and reduced hemodynamic and mechanical stress. However, these pathways are closely interdependent, making it difficult to isolate the contribution of BW reduction from the broader pharmacological effects of GLP-1 receptor agonism.

SELECT is particularly informative because semaglutide reduced MACE in individuals with overweight or obesity and established CVD but without diabetes (14). The absence of diabetes indicates that improved glycemic control is not required for CV benefit in this population. Nevertheless, SELECT does not establish that the benefit is independent of weight loss, because semaglutide also produced sustained reductions in BW and WC together with changes in several other CV risk factors. Thus, the trial supports CV effects beyond glucose lowering but does not allow the relative contributions of WR, BP, inflammation, vascular effects, or other pathways to be determined.

Exploratory mediation analyses further illustrate this uncertainty. In a *post-hoc* analysis of LEADER and SUSTAIN-6, changes in HbA1c were estimated to mediate approximately 25% and 26% of the kidney effect, respectively, while systolic BP accounted for approximately 9% and 22%. BW accounted for only approximately 9% of the effect in LEADER and showed no apparent mediation in SUSTAIN-6 (79). These analyses were exploratory, confidence intervals were imprecise, and the kidney composites were largely driven by albuminuria; therefore, the estimated mediation proportions should not be interpreted as definitive causal fractions of the treatment effect.

The SELECT kidney analysis provides a different but similarly cautious perspective (15). Formal mediation analysis of the main composite kidney endpoint was not adequately powered. Exploratory analyses showed little correlation between individual changes in eGFR and changes in BW, systolic BP, or HbA1c. A separate mediation analysis of eGFR change suggested that approximately 81% of the treatment-associated difference in eGFR at 104 weeks might be attributable to weight change; however, the estimate was highly imprecise (95% CI 41.3-120%) and should therefore be interpreted cautiously. Taken together, available mediation analyses demonstrate that estimates depend substantially on the population, endpoint, statistical model, and mediator considered and do not permit definitive separation of weight-dependent from weight-independent kidney effects.

Potential weight-independent pathways remain biologically plausible. GLP-1 receptor agonism has been associated with natriuresis, alterations in kidney hemodynamics, anti-inflammatory and antioxidative effects, improvement in endothelial function, and modulation of vascular and fibrotic pathways (36–39). However, much of the evidence supporting direct tissue effects derives from experimental or mechanistic studies, and their quantitative contribution to the clinical CV and kidney benefits observed in large outcome trials remains uncertain. Such mechanisms should therefore be regarded as complementary hypotheses rather than established explanations of outcome benefits.

The comparison with tirzepatide further demonstrates why the magnitude of weight loss should not be used as a surrogate for the magnitude of cardiorenal protection. Tirzepatide generally produces greater reductions in BW than single GLP-1 RAs in metabolic and obesity trials, yet SURPASS-CVOT demonstrated non-inferiority rather than superiority to dulaglutide for MACE despite greater metabolic effects (22). Similarly, although SUMMIT demonstrated clear clinical benefit in obesity-related HFpEF, the composite benefit was driven primarily by fewer worsening HF events rather than a standalone reduction in CV mortality (21). Dedicated kidney outcome evidence for tirzepatide remains unavailable. Overall, the available evidence supports a multifactorial model in which WR contributes importantly to cardiorenal benefit but acts together with changes in BP, glycemia, visceral adiposity, inflammation, vascular function, and potentially direct organ effects.

### 5.3 Combination therapy within multimodal CKM syndrome management

Contemporary management of CKM syndrome increasingly relies on complementary, phenotype-directed therapies rather than on a single pharmacological class. The 2026 AHA/ACC/ADA/ASN Guideline reflects this approach and supports the use of SGLT2 inhibitors, GLP-1-based therapies, or both in appropriate patients with T2D and CV or kidney risk (3). In patients with T2D and CKD, SGLT2 inhibitors remain a central component of kidney-protective therapy, while GLP-1-based treatment may be added according to residual CV and kidney risk, obesity, glycemic status, albuminuria, and other CKM syndrome characteristics.

There is a strong biological rationale for combining SGLT2 inhibitors with incretin-based therapies because their mechanisms overlap only partially. SGLT2 inhibition promotes natriuresis and osmotic diuresis, restores tubuloglomerular feedback, reduces intraglomerular pressure, and produces favorable systemic and cardiac hemodynamic effects. GLP-1 receptor agonism provides substantial effects on BW and glycemic control and may additionally improve BP, systemic inflammation, vascular function, and other metabolic determinants of CV and kidney risk. Thus, combination therapy has the potential to target complementary components of CKM syndrome pathophysiology rather than simply intensifying a single pathway.

Clinical evidence increasingly supports compatibility of these approaches, although evidence of true additivity remains less definitive. In a systematic review and meta-analysis of randomized GLP-1 RA outcome trials, Neuen et al. found that GLP-1 RAs reduced MACE irrespective of baseline SGLT2 inhibitor use, with no evidence of treatment-effect heterogeneity; kidney outcomes and eGFR slope showed a similarly consistent pattern (80). These findings support preservation of GLP-1 RA benefit during background SGLT2 inhibitor treatment and are consistent with independent or complementary effects. However, baseline SGLT2 inhibitor treatment was not randomized and only a minority of participants were receiving an SGLT2 inhibitor, so the analyses do not directly quantify incremental benefit from combination treatment (80, 81).

FLOW provides additional evidence in patients with T2D and CKD. In a prespecified analysis, 550 participants were receiving an SGLT2 inhibitor at baseline. For the primary composite outcome, the HR for semaglutide versus placebo was 1.07 (95% CI 0.69-1.67) among participants receiving an SGLT2 inhibitor and 0.73 (95% CI 0.63-0.85) among those not receiving one; the interaction was not statistically significant (P for interaction=0.109). Treatment effects on eGFR slope, MACE, and all-cause mortality were likewise not significantly modified by baseline SGLT2 inhibitor use (82). The wide confidence interval in the SGLT2 inhibitor subgroup and the relatively small subgroup size limit the ability to determine the magnitude of incremental kidney benefit from combination therapy.

Consequently, available randomized-trial subgroup data support compatibility and potential complementarity of GLP-1 RAs and SGLT2 inhibitors but do not yet establish the precise magnitude of additive CV or kidney protection. Evidence is even more limited for tirzepatide: some SURPASS trials permitted background SGLT2 inhibitor therapy, but no dedicated CV or kidney outcome trial has established the incremental organ-protective benefit of adding tirzepatide to an SGLT2 inhibitor. The GLP-1 RA combination evidence should therefore not be extrapolated quantitatively to dual GIP/GLP-1 receptor agonism.

### 5.4 Safety, tolerability, and considerations in vulnerable populations

As incretin-based therapies are increasingly used for long-term management of T2D, obesity, CVD, and CKD, their safety profile should be considered alongside cardiorenal benefits. GI adverse events remain the most frequent tolerability limitation of both GLP-1 RAs and tirzepatide. Nausea, vomiting, diarrhea, constipation, and abdominal symptoms are generally mild to moderate and transient and occur most frequently during treatment initiation and dose escalation (83, 84). In pooled STEP 1–3 analyses of semaglutide 2.4 mg, GI adverse events led to permanent treatment discontinuation in 4.3% of participants, and most events occurred during or shortly after dose escalation (85). In the SURMOUNT program, GI adverse events with tirzepatide were likewise concentrated during dose escalation, with 1.0-10.5% of tirzepatide-treated participants discontinuing treatment because of GI events depending on the trial and regimen (86). These findings emphasize that pharmacological efficacy and treatment persistence are distinct considerations in routine practice.

Delayed gastric emptying is an expected pharmacological effect of GLP-1 receptor agonism and may contribute to upper GI symptoms. Its clinical relevance varies substantially between individuals and may be greater in patients with pre-existing GI motility disorders or marked symptoms during dose escalation (83). Gradual dose escalation and individualized management of GI adverse effects are therefore important components of treatment tolerability.

Gallbladder and biliary events represent another clinically relevant safety consideration. A meta-analysis of 76 randomized trials involving more than 100, 000 participants found that GLP-1 RA therapy was associated with an increased risk of gallbladder or biliary disease (RR 1.37, 95% CI 1.23-1.52), with stronger associations in weight-management trials, at higher doses, and with longer treatment duration (87). Meta-analytic data for tirzepatide have also identified increased risks of gallbladder or biliary disease (RR 1.52, 95% CI 1.17-1.98) and cholelithiasis (RR 1.67, 95% CI 1.14-2.44) (88). Rapid or substantial weight loss itself may contribute to gallstone formation, while incretin-based pharmacology may additionally influence gallbladder motility; absolute event rates nevertheless remain relatively low.

Acute pancreatitis is a serious but rare event that warrants clinical vigilance. Current randomized-trial meta-analyses have not demonstrated a statistically significant increase in acute pancreatitis with semaglutide (OR 0.70, 95% CI 0.50-1.20) or with tirzepatide compared with placebo or active comparators, although the absolute number of events is small and estimates remain imprecise (89, 90). These data are reassuring but cannot exclude a very small risk.

A further semaglutide-specific consideration concerns diabetic retinopathy. In SUSTAIN-6, diabetic retinopathy complications occurred more frequently with semaglutide than with placebo (9). Subsequent analyses suggested that the excess events were concentrated predominantly among participants with pre-existing diabetic retinopathy, poor baseline glycemic control, insulin treatment, and a large and rapid initial reduction in HbA1c. This pattern is consistent with the recognized phenomenon of early worsening of diabetic retinopathy following rapid improvement in glycemic control rather than establishing generalized direct retinal toxicity (91). Patients with advanced pre-existing retinopathy and markedly elevated HbA1c may therefore warrant particular attention when treatment produces rapid glycemic improvement.

Substantial WR also raises specific considerations regarding body composition and nutritional status. Both semaglutide and tirzepatide reduce not only fat mass but also some lean body mass during weight loss (92–94). Loss of lean mass should not be considered synonymous with sarcopenia, which additionally requires impairment of muscle strength and/or physical performance. Available clinical data do not establish that GLP-1-based therapy independently increases the incidence of sarcopenia in the general population, but uncertainty is greater in older and frail individuals, particularly those with low baseline muscle reserves, CKD, HF, multimorbidity, or limited mobility. Marked appetite suppression and persistent GI adverse effects may further compromise energy, protein, micronutrient, or fluid intake. In these vulnerable patients, monitoring of weight-loss trajectory, nutritional intake, and muscle function, together with adequate protein intake and resistance exercise where feasible, may be particularly relevant (92–96).

### 5.5 Next-generation incretin agonists and future therapies

The therapeutic landscape of incretin-based pharmacotherapy continues to evolve beyond single GLP-1 receptor agonism and dual GIP/GLP-1 receptor agonism. Retatrutide, a single-molecule agonist targeting GLP-1, GIP, and glucagon receptors, represents one of the most advanced examples of next-generation multi-receptor therapy. Early and phase 3 studies have demonstrated marked reductions in BW together with substantial metabolic improvements, suggesting that triple-receptor agonism may further expand the efficacy of incretin-based treatment (97–99).

However, the extent to which these pronounced metabolic effects translate into CV and kidney protection remains uncertain. The ongoing TRIUMPH-Outcomes trial is evaluating major CV and kidney outcomes in individuals with obesity and established CVD and/or CKD (100). These results will be important in determining whether greater weight-loss efficacy with triple agonism is accompanied by additional hard cardiorenal benefit. Until such outcome data are available, retatrutide should be regarded as an emerging therapy rather than as part of the established cardiorenal evidence base reviewed for GLP-1 RAs and tirzepatide.

## 6 Study limitations

Several limitations of this review should be acknowledged. First, as a narrative review, this article did not employ a predefined systematic review protocol, formal study-selection procedures, risk-of-bias assessment, or quantitative evidence synthesis. Consequently, potential selection bias in the identification and interpretation of the available literature cannot be excluded. Second, the quantity, quality, and consistency of evidence vary across the topics discussed, with some areas supported by large outcome RCTs and others relying on secondary or exploratory analyses, subgroup comparisons, mechanistic studies, or still-evolving evidence.

The persistence of CV and kidney protection with incretin-based therapies beyond the follow-up periods of available RCTs, generally limited to several years, also remains uncertain. Treatment adherence and persistence in routine practice may differ from those observed in clinical trials because of tolerability, cost, access, treatment burden, and patient preference. Evidence remains limited in several populations, including patients with advanced CKD or receiving dialysis, older or frail adults, and patients with HFrEF, heart failure with reduced ejection fraction who were not selected on the basis of coexisting obesity.

Finally, the therapeutic landscape is evolving rapidly, and newly emerging clinical trial, guideline, regulatory, and pharmacovigilance data may modify some of the interpretations presented in this review. Accordingly, this article should be regarded as a clinically oriented synthesis of current evidence rather than a source of definitive treatment recommendations. Its primary purpose is to integrate the available evidence, clarify differences in its maturity across outcomes and agents, identify unresolved questions, and highlight priorities for future clinical and translational research.

## 7 Conclusions and future directions

Incretin-based therapies have substantially transformed the management of T2D, obesity, and CKM syndrome by extending therapeutic goals beyond glycemic control and WR toward CV and kidney risk reduction The main conclusions derived from clinical and mechanistic studies on CV and kidney effects of single GLP-1RAs and tirzepatide are summarized in **Figures 1** and **2**, respectively. However, these therapies should not be considered interchangeable, because the strength and maturity of outcome evidence differ substantially across individual agents and clinical phenotypes.

**Figure 1:**
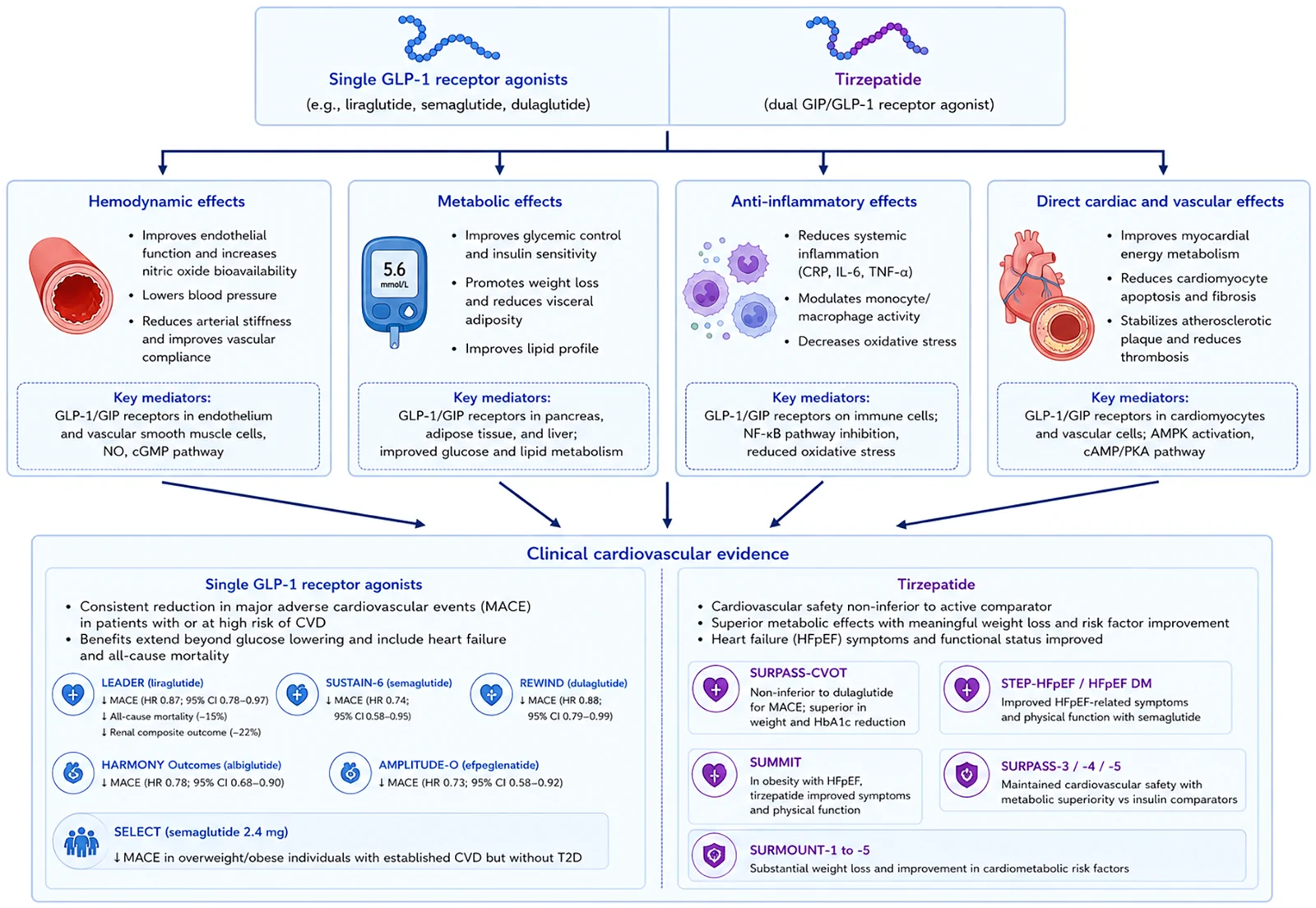
GLP-1 receptor agonists and the dual GIP/GLP-1 receptor agonist tirzepatide as cardioprotective agents. Schematic overview of the principal mechanisms and clinical evidence supporting CV effects of single GLP-1RAs and tirzepatide. Potential cardioprotective pathways include favorable hemodynamic effects, improvements in glycemic control, insulin sensitivity, BW and lipid metabolism, attenuation of systemic inflammation and oxidative stress, and potential direct cardiac and vascular actions. CV outcome trials with single GLP-1RAs, including LEADER, SUSTAIN-6, REWIND, HARMONY Outcomes, and AMPLITUDE-O, demonstrated reductions in MACEs in patients with T2D and established CVD or elevated CV risk. SELECT further demonstrated CV benefit with semaglutide in individuals with overweight or obesity and established CVD without T2D. For tirzepatide, SURPASS-CVOT established CV safety relative to dulaglutide, while SUMMIT demonstrated clinically relevant benefits in patients with obesity and HFpEF. Together, these findings support a transition from a predominantly glucose-centered view of incretin-based therapies toward a broader cardiometabolic and organ-protective perspective. AMPK, AMP-activated protein kinase; cAMP, cyclic adenosine monophosphate; BW, body weight; CKD, chronic kidney disease; CRP, C-reactive protein; CV, cardiovascular; CVD, cardiovascular disease; GIP, glucose-dependent insulinotropic polypeptide; GLP-1, glucagon-like peptide-1; GLP-1RA, glucagon-like peptide-1 receptor agonist; HFpEF, heart failure with preserved ejection fraction; IL-6, interleukin-6; MACE, major adverse cardiovascular events; NF-κB, nuclear factor kappa B; NO, nitric oxide; T2D, type 2 diabetes; TNF-α, tumor necrosis factor alpha.

**Figure 2:**
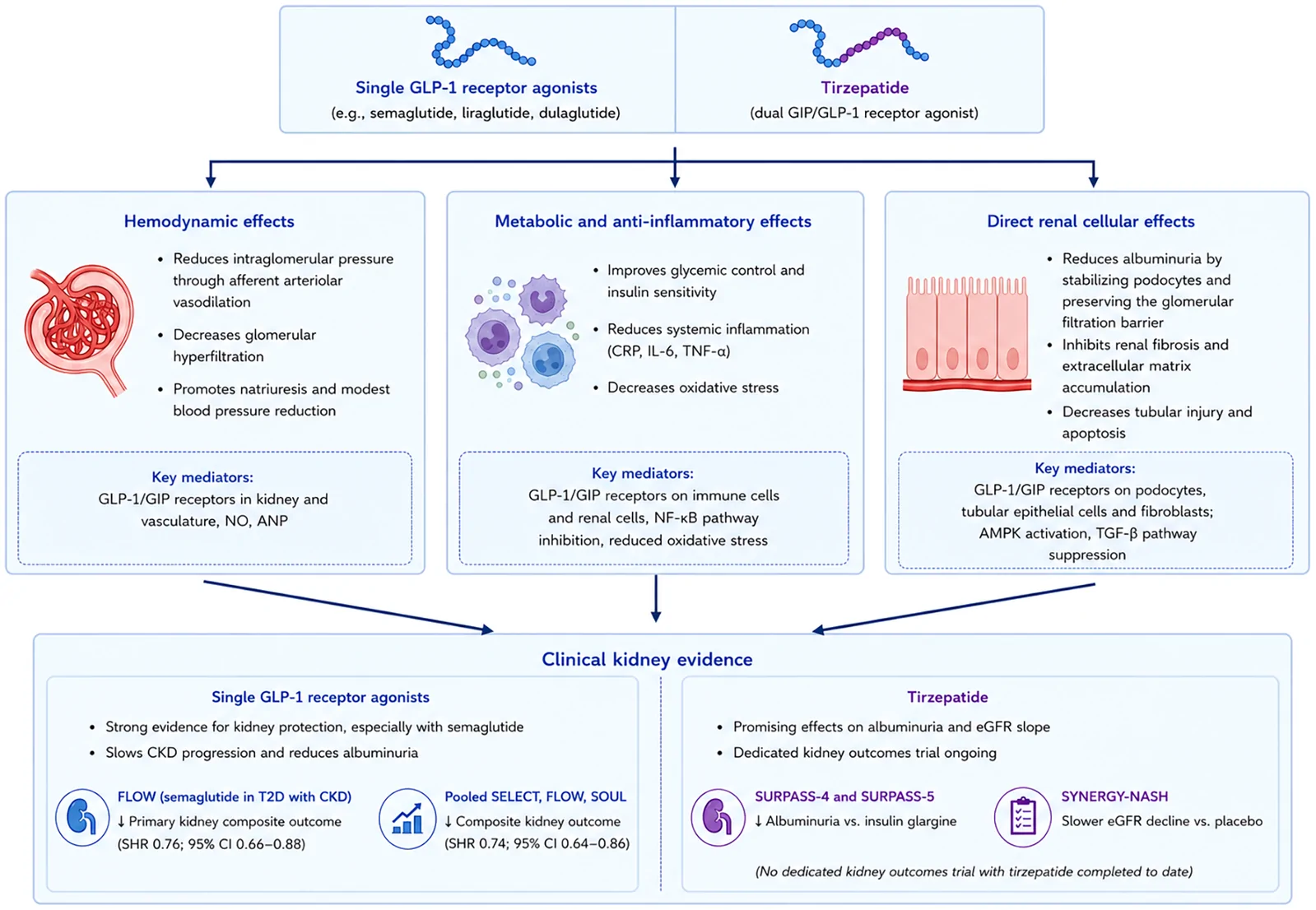
GLP-1 receptor agonists and the dual GIP/GLP-1 receptor agonist tirzepatide as nephroprotective agents. Schematic representation of the principal mechanisms potentially contributing to kidney protection with single GLP-1RAs and tirzepatide. These effects involve hemodynamic pathways, including modulation of intraglomerular pressure, glomerular hyperfiltration, and natriuresis; metabolic and anti-inflammatory actions associated with improved glycemic control, reduced systemic inflammation, and oxidative stress; and potential direct renal cellular effects influencing albuminuria, fibrosis, and tubular injury. Clinical evidence supports kidney protection with GLP-1RAs, particularly semaglutide, as demonstrated in FLOW and further supported by pooled analyses of SELECT, FLOW, and SOUL. Tirzepatide has shown favorable effects on albuminuria and eGFR trajectories; however, evidence from dedicated RCTs with hard kidney outcomes remains limited. The proposed mechanisms should be interpreted as complementary and may include both direct and indirect pathways. ANP, atrial natriuretic peptide; AMPK, AMP-activated protein kinase; CKD, chronic kidney disease; eGFR, estimated glomerular filtration rate; GIP, glucose-dependent insulinotropic polypeptide; GLP-1, glucagon-like peptide-1; GLP-1RA, glucagon-like peptide-1 receptor agonist; NO, nitric oxide; RCT, randomized controlled trial; SHR, subdistribution hazard ratio; T2D, type 2 diabetes.

Current treatment selection should therefore consider both the predominant CKM syndrome phenotype and the type of outcome evidence available. CV outcome benefits have been established for several GLP-1 RAs in patients with T2D and elevated CV risk, while dedicated evidence for slowing clinically meaningful CKD progression is currently strongest for semaglutide, based on FLOW and supported by subsequent pooled analyses across diverse populations with CKM syndrome (16, 18). These data provide a particularly strong rationale for semaglutide when reduction of CKD progression is a major therapeutic priority.

Tirzepatide provides particularly strong metabolic efficacy, including substantial reductions in HbA1c and BW, together with an expanding CV evidence base. SURPASS-CVOT demonstrated non-inferiority to dulaglutide for MACE in patients with T2D and established atherosclerotic CVD (22), whereas SUMMIT provided particularly encouraging evidence in patients with obesity-related HFpEF by reducing worsening HF events and improving health status (21). By contrast, kidney evidence for tirzepatide remains less mature and is derived predominantly from secondary and *post-hoc* analyses rather than from a dedicated kidney outcome trial (23).

Accordingly, the clinical value of incretin-based therapies is best interpreted within a phenotype-oriented framework rather than according to metabolic efficacy alone. In patients with established atherosclerotic CVD, CKD, obesity, or obesity-related HFpEF, the choice of therapy should reflect the clinical outcome that is most important to modify, the maturity of evidence supporting that outcome, patient characteristics and comorbidities, tolerability, and the role of complementary guideline-directed therapies. Contemporary CKM syndrome management increasingly incorporates combinations of therapies with complementary mechanisms, particularly SGLT2 inhibitors and GLP-1-based therapies; available evidence suggests that GLP-1 RA benefits are preserved during background SGLT2 inhibitor treatment, although the magnitude of incremental benefit attributable specifically to combination therapy remains uncertain (3, 80–82).

Important evidence gaps remain. Dedicated kidney outcome trials are still needed for tirzepatide, and further studies should clarify optimal sequencing and combination strategies, including dual GIP/GLP-1 receptor agonism and nonsteroidal mineralocorticoid receptor antagonists (nsMRA), with attention to long-term safety, cost-effectiveness, treatment burden, and equitable access (25, 101–105). Additional evidence is also required in patients with advanced CKD, those receiving dialysis, older or frail individuals, and people with T1D, in whom both therapeutic benefit and safety may differ from those observed in the populations enrolled in major RCTs (106, 107).
